# The Sufferings of Christ

**Published:** 1888-03-03

**Authors:** 


### WORDS OF CONSOLATION.

[Communications for this column should be addressed "Chaplain," care of the Editor of The Hospital, who will gratefully welcome any suggestions or inquiries from matrons, nurses, and the staff generally.]

### LXVIII.—The Sufferings of Christ.

*For Reading to the Sick.*

It has been said: "To make the suffering complete there was not only a rejection of the Godhead of our Blessed Lord, but a rejection of the manhood. When Pilate brought Him forth, and said, 'Behold the Man!' this was the last appeal to human compassion on behalf of the helpless misery of His humanity. 'Not this man, but Barabbas,' was the answer. 'What shall I do then with Jesus, which is called Christ?' Then there arose throughout the court, and along the crowded streets of the city, the cry, 'Crucify! crucify!'—not crucify Him, but more likely, according to the original Greek, it is simply 'Crucify!' as though no person is recognised. His very humanity is disowned. His very existence as a Being was cast out from mankind, and the language of prophecy was completely fulfilled. He became to them 'a worm, and no man,' the outcast of the people—despised, rejected altogether; no man cared for His soul."

Desertion, desolation, being forsaken! Do not such terms acquire a deeper meaning than is generally accorded to them in connexion with the crucifixion, when we regard them as pointing to the essence of the second death. What is that from which infinite Love came to redeem us but rejection by God? What is it, or what will it be in the case of the wicked but for humanity to be lost, for the persons of the impenitent to be at last disowned by their Maker? That will be for sin to bring forth death in all its frightful reality, in which at last there can be no form, nor comeliness, no beauty to be desired; the creature still abiding, yet as it were without form and void; while darkness settles down upon the face of the great deep, without the good Spirit being sent any more to breathe upon the troubled waters and make them instinct with life again.

There is much in Holy Scripture to teach us what joy may be in store for us in heaven through the preservation of our identity. "Jesus Christ is the same yesterday, to-day, and for ever" (Heb. xiii. 8). You look forward as a member of His body to being more than saved. You hope to be known, recognised, loved as the person. You were here, but without sin. "I know my sheep, and am known of mine" (John x. 14). Surely, even as He knows them, they will then be known to one another more intimately than they ever could be here. It may be that the Christian name given us at the font shall, instead of being forgotten, be preserved in the society of heaven. Certain it is that where love reigns supreme—where all suspicions, misunderstandings, and prejudices are banished for ever, there too must hearts attain a depth of intimacy in the perfected communion of the saints which is not possible here. And this will be the fruit of the Cross. But in hell must be found the extreme contradiction of this blessedness. There seem to be no names in hell except the names of sins. The very identity of the lost seems to be merged in their ruin. They seem to be forgotten, to be out of mind, to be given over to a forsaking. They look for some one to have pity; but no one careth for their souls. Yea, the only persons, feelings, things that cling to them are those they would so willingly be forsaken by. The consolations of the Cross are by no means depreciated by looking steadily at the dark side of the picture as well as at the other. You do not realize the value of salvation unless you meditate upon the death from which you are redeemed.

Singing in the ears, or *tinnitus aurium*, is sometimes the accompaniment of general disturbance of the constitution, sometimes the result of direct irritation. Roosa and Kramer tell of two cases of suicide on account of it, and Sauvage mentions an instance of a musician who heard, in addition to every note he played, a second inharmonious sound, so that he was compelled to give up his occupation.

"The giving of money without thought is continually mischievous, but the invective of the economist against indiscriminate charity is idle if it be not coupled with pleading for discriminate charity, and, above all, for that charity which discerns the uses that people may be put to, and helps them by setting them to work in those services. That is the help beyond all others—find out how to make useless people useful, and let them earn their money instead of begging it."—*Ruskin.*

